# Layer-by-Layer Assembly of Polyelectrolyte Multilayer onto PET Fabric for Highly Tunable Dyeing with Water Soluble Dyestuffs

**DOI:** 10.3390/polym9120735

**Published:** 2017-12-20

**Authors:** Shili Xiao, Pengjun Xu, Qingyan Peng, Jiali Chen, Jiankang Huang, Faming Wang, Nuruzzaman Noor

**Affiliations:** 1 Key Laboratory of Green Processing and Functional Textiles of New Textile Materials, Ministry of Education, Wuhan Textile University, Wuhan 430073, China; 15629184851@163.com (Q.P.); tears0918@163.com (J.C.); 13545350343@163.com (J.H.); 2 Faculty of Clothing and Design, Minjiang University, Fuzhou 350108, Fujian, China; xupj@mju.edu.cn; 3 Institute of Textiles and Clothing, The Hong Kong Polytechnic University, Hung Hom, Kowloon, Hong Kong, China; nuru.noor@polyu.edu.hk

**Keywords:** polyethylene terephthalate fabric, polyelectrolyte multilayer, layer-by-layer, self-assembly, water soluble dyestuff

## Abstract

Poly(ethyleneterephthalate) (PET) is a multi-purpose and widely used synthetic polymer in many industrial fields because of its remarkable advantages such as low cost, light weight, high toughness and resistance to chemicals, and high abrasion resistance. However, PET suffers from poor dyeability due to its non-polar nature, benzene ring structure as well as high crystallinity. In this study, PET fabrics were firstly treated with an alkaline solution to produce carboxylic acid functional groups on the surface of the PET fabric, and then was modified by polyelectrolyte polymer through the electrostatic layer-by-layer self-assembly technology. The polyelectrolyte multilayer-deposited PET fabric was characterized using scanning electron microscopy SEM, contact angle, Fourier transform infrared (FTIR) and X-ray photoelectron spectroscopy (XPS). The dyeability of PET fabrics before and after surface modification was systematically investigated. It showed that the dye-uptake of the polyelectrolyte multilayer-deposited PET fabric has been enhanced compared to that of the pristine PET fabric. In addition, its dyeability is strongly dependent on the surface property of the polyelectrolyte multilayer-deposited PET fabric and the properties of dyestuffs.

## 1. Introduction

As a main component of polyester, poly(ethyleneterephthalate) (PET) has been widely used in household, packaging, construction and textile industries because of its desirable properties such as low cost, light weight, high toughness, resistance to chemicals, and high abrasion resistance [1,2,3]. In particular, PET ‘polyester’ fibers have gained growing attention in textile industry due to its numerous advantages including abundance of raw materials, lower cost as opposed to natural textiles and the possibility of recycling [4,5]. However, PET fabrics suffer from poor dyeability due to its non-polar nature, compact molecular structure as well as high crystallinity, which limit the accessibility of dye molecules [2]. Thus, dyeing PET with water insoluble dyes, namely disperse dyes to impart PET fiber coloration, is the main method. Dyeing PET with disperse dyes is usually carried out at high temperature (above 100 °C) and high pressure, where dispersed dye particles in aqueous solution is transferred from bulk solution to the fiber surface, and then diffused from the surface into the interior of the fiber. During this process, solvents and carriers are widely used as a means of accelerating dyeing rate, improving dye uptake and lowering dyeing temperature [6,7,8]. However, these additives have raised serious problems, like toxicity, unpleasant odor, and environmental contamination [9]. Thus, much work needs to be done in order to improving the dyeability of PET, lowing the cost and energy and mediating adverse effects during dyeing process. 

For this purpose, numerous approaches have been developed from both scientific and commercial points of view [8,10,11,12]. Among these, enhancing PET’s functional characteristics and improving its hydrophilic property through surface modification has gained particular attention [2,4,10,13]. For instance, Shahidi and co-workers [14] coated platinum nano-layers onto a polyester fabric using DC magnetron sputtering to improve the dyeability of polyester fabrics. Dyeing experiments confirmed that polyester fabrics with platinum nano-layers showed improved dyeing property; the relative color strength and fastness properties of dyed samples improved significantly, especially for the natural dyestuffs. Alternatively, Salem [4] modified plasma pre-treated PET fabrics with poly(diallydimethylammonium chloride) (PDADMAC), a kind of polyelectrolyte that possesses a charged quaternary ammonium group in each monomer unit providing the polymer with high water solubility and high cationic strength. Evidently, the water absorptivity of PET fabric was greatly enhanced after plasma treatment and acid dyes were fixed onto the surface of PDADMAC modified PET fabrics via electrostatic interactions, whereas no color was observed for the unmodified fabrics. Moreover, other surface modification methods like surface oxidation, surface hydrolysis, surface grafts and ionized modification have been intensively used to introduce hydrophilic groups such as –COOH, –OH –NH_2_ and –SO_3_H groups etc. to the PET macromolecules to make the PET fabric hydrophilic [15,16,17]. Although enhanced hydrophilicity has been obtained, these methods are either energy intensive or involve complex chemical reactions. Therefore, searching for a simple strategy to modify the surface of PET fabric with tailored surface property in moderate condition still remains a challenge. 

Layer-by-layer (LbL) electrostatic self-assembly technique has received immense scientific and technological interest as a facile and powerful approach to preparing materials with tailored properties [18,19,20]. In the LbL assembly procedure, oppositely charged polyelectrolytes are alternately deposited on a pre-charged substrate to generate a nanometer-scale polyelectrolyte film on its surface. During this process, the thickness, morphology and composition of the covering film can be controlled by varying the number of deposition cycles and by tuning the composition of the layers, which makes the technique highly attractive in preparing surface-mediated functional materials [21,22]. A series of nanoparticles, polymers, and biomolecules have been successfully assembled onto various substrates, which have found wide application in sensors [23,24], environmental remediation [25,26], drug delivery and tissue engineering [27,28,29]. Taking advantage of LbL technology in surface modification of materials, Chen and McCarthy [30] systematically investigated the deposition process of polyelectrolytes onto PET film using X-ray photoelectron spectroscopy technology and contact angle measurement. It proved that the multilayer nanofilm has been successfully built up on PET film, the polyelectrolyte layers were stratified and the wettability of the multilayer assemblies was largely controlled by the identity of the outermost polyelectrolyte layer. According to these works, it is reasonable to hypothesize that depositing polyelectrolytes onto PET surface using the LbL technology could be a simple and effective strategy to modify the PET fabric for enhanced coloration.

In the present study, polyelectrolyte multilayer-coated PET fabrics were developed using the LbL technology. In this approach, the PET fabric was first pretreated with alkaline solution to produce a carboxyl-enriched surface, which was negatively charged. Then, positively charged poly(diallyldimethylammonium chloride) (PDADMAC) and negatively charged poly(acrylic acid) (PAA) were alternately assembled onto the pre-treated PET fabric through electrostatic interaction to get PAA/PDADMAC multilayer-coated PET fabric. The scanning electron microscopy (SEM), Fourier transform infrared (FTIR) spectroscopy and X-ray photoelectron spectroscopy (XPS) were utilized to characterize the morphology and composition of the PAA/PDADMAC multilayer-coated PET fabric. The contact angle of PET fabrics before and after surface modification was measured. The dyeing property of multilayer-coated PET fabric was examined using different water soluble dyestuffs. We show that the developed PAA/PDADMAC multilayer-coated PET fabric display a significantly enhanced dyeability to model dyestuffs. To the best of our knowledge, this is the first report related to the surface modification of PET fabric using the LbL assembly approach and systematic investigation of dyeability of modified PET fabric to water soluble dyestuffs. The major advantage of this approach lies in the feasibility to tune the surface property of PET fabric, thereby mediating its adsorption capability to water soluble dyestuffs. To the best of our knowledge, this is the first report related to the surface modification of PET fabrics using the LbL assembly approach and also, this study presents a systematic investigation of dyeability of LbL-modified PET fabrics to water soluble dyestuffs. The major advantage of this approach lies in the feasibility to tune the surface property of PET fabric, thereby mediating its adsorption capability to water soluble dyestuffs.

## 2. Materials and Methods

### 2.1. Materials

Poly(diallyldimethylammonium chloride) (PDADMAC, average *M_w_* = 100,000–200,000, 20 wt %) and poly(acrylic acid) (PAA, average *M_w_* = 240,000, 25 wt %) were bought from *Aldrich*. NaCl (Analytical reagent, 99.5%) and NaOH (Guaranteed reagent, 97%) were purchased from Sinopharm Chemical Reagent Co., Ltd. (Shanghai, China). Four commercial dyes, namely methylene blue, acid red, methyl blue, and brilliant green were sourced from Shanghai Jingchun Reagent Co., Ltd. (Shanghai, China). PET plain weave fabric (fabric areal weight: 145 g/m^2^) was kindly supplied by Jiangnan Co., Ltd. (Wuhan, China).

### 2.2. Preparation of Polyelectrolyte Multilayer-Deposited PET Fabric

Before deposition the polyelectrolyte multilayers, the PET fabric was pre-treated with 0.2 M NaOH solution at 70 °C for 1 h to produce a carboxyl-enriched, negatively charged surface. The pre-treated PET fabric was thoroughly rinsed and air dried at room temperature. The negatively charged PET fabric enabled the deposition of positively charged PDADMAC as the first layer. PDADMAC and PAA layers were LbL-assembled onto the negatively charged PET fabric alternatively to create a hydrophilic PET fabric. During complex formation by immersing in solution, the oppositely charged polymer chains will likely interpenetrate; whilst there will predominantly be an excess of the last added polymer at the surface, it is unlikely to be exclusive—there will be some intermixing. PDADMAC and PAA solutions used for deposition were prepared in weakly saline water (containing 0.5 M NaCl) with a concentration of 2 mg/mL. The pH value of both solutions was adjusted to 3.5 using either 1 M HCl or 1 M NaOH. The negatively charged PET fabric (5 cm × 5 cm) enabled the deposition of positively charged PDADMAC as the first layer. In a typical procedure, PET fabric was first immersed into PDADMAC solution for 5 min, followed by rinsing with water three times (each rinsing step took 3 min). Then, the substrate was immersed in the negatively charged PAA solution for 5 min, followed by similar rinsing steps in water. The immersion/rinsing cycles were repeated until the desired number of bilayers was achieved. Generally, the low molar mass counter ions, Na^+^ and Cl^−^, originating from PAA and PDADMAC are liberated during complex formation and removed during the washing procedures executed after each immersion step. The completeness of counterion removal depends, for example, on the intensity of the washing process, the chemical structure of the polymer chains, complementarity of the charge density etc. The polyelectrolyte multilayers thus obtained were denoted as (PDADMAC/PAA)*_n_*. Each assembly of a PAA layer followed by a PDADMAC layer was defined as a bilayer in our naming system, although the first layer of PDADMAC was regarded as a base layer and was not include in the bilayers in our naming system. The polyelectrolyte multilayer-deposited PET fabrics with different bilayers ((PDADMAC/PAA)*_n_*-PET) were finally dried at room temperature and stored in desiccators before use. 

### 2.3. Characterization

The surface morphologies of PET fabric and polyelectrolyte multilayer-deposited fabrics were observed using scanning electron microscopy (SEM) (*JSM-5600 LV*, JEOL, Ltd., Tokyo, Japan) with an operating voltage of 20 kV. Contact angle measurements were carried out with *FM40 Easy Drop* contact angle tester (KRÜSS GmbH, Hamburg, Germany). FTIR spectra of samples were recorded on *VERTEX 70* spectrometer (Bruker Corp., Billerica, MA, USA) at a wavenumber range of 4000–500 cm^−1^ under ambient conditions. X-ray photoelectron spectroscopy (XPS) measurements were carried out on a *Thermo Scientific Escalab 250Xi XPS system* (Thermo Fisher Scientific Inc., Waltham, MA, USA) with Al Kα X-ray source and a charge neutralizer.

### 2.4. Dyeing of Polyelectrolyte Multilayer-Deposited PET Fabric

**Figure 1:**
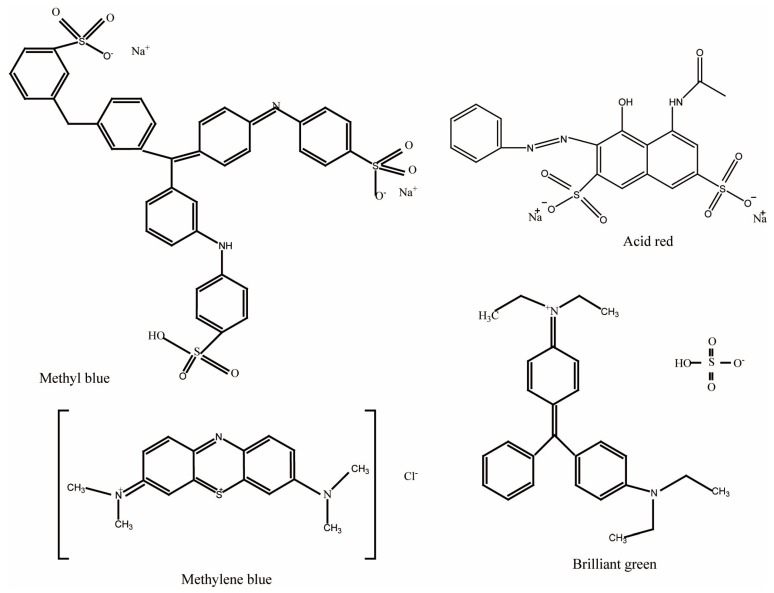
Molecular structure of dyes.

PET fabrics with and without polyelectrolyte multilayer deposition were dyed with methylene blue, acid red, methyl blue and brilliant green, respectively. Normally, charged dyes penetrate into the LbL-formed multistructure via electrostatic interaction—there is usually no bond formation. The concentration of each dye solution was 10 g/L. Figure 1 shows the chemical structures of used dyes. Polyelectrolyte multilayer-deposited PET fabric was immersed in dye solution at room temperature for 2 h under gentle stirring. The weight ratio of PET fabric to the dye solution was 1:100. The dyed PET fabrics under different conditions were individually tested for their color strength with a colorimeter (COLOR I7, X-Rite Inc., Grand Rapids, MI, USA). The color strength (*K*/*S*) values of the dyed fabrics were determined from the wavelength of minimum reflection by using Equation (1):

(1) $$\frac{K}{S}=\frac{{\left(1-R\right)}^{2}}{2R}$$

where *R* refers to the reflection value of the sample, *S* refers to the scattering value and *K* refers to the sample absorbance. 

### 2.5. Colorfastness Test to Washing

Washing fastness tests were performed in accordance with AS 2001.4.15-2006. The methylene blue dyed PET fabric deposited with 2.5 bilayers of polyelectrolyte multilayers were washed for 30 min at 40 °C in the presence of ECE reference detergent (i.e., BS EN ISO/SDC ECE Phosphate Reference Detergent B). The CIE Lab color coordinate values (L*, a*, and b*) for each samples were measured before and after washing, where L* represents the lightness/darkness, a* represents the red or green Chroma, and b* represents the chromaticity coordinate for yellow/blue. The color difference (∆*E*) was calculated using Equation (2):

(2) $$\Delta E=\sqrt{{\left(L_{2}^{*}-L_{1}^{*}\right)}^{2}+{\left(a_{2}^{*}-a_{1}^{*}\right)}^{2}+{\left(b_{2}^{*}-b_{1}^{*}\right)}^{2}}$$

### 2.6. Measurement of Fastness to Light

The methylene blue dyed PET fabric deposited with 2.5 bilayers of polyelectrolyte multilayers were exposed to high intensity simulated sunlight for 10 min, 20 min, 40 min and 60 min, respectively inside the homemade Sun test instrument (the power of simulated sunlight is 500 W). The CIE Lab color coordinated values of samples exposed with sunlight for different time were measured by the colorimeter (COLOR I7, X-Rite Inc., Grand Rapids, MI, USA). Color changes were determined with the ∆*E* value, which can be calculated according to Equation (2).

## 3. Results and Discussion

### 3.1. Preparation of Polyelectrolyte Multilayer-Deposited PET Fabric

**Figure 2:**
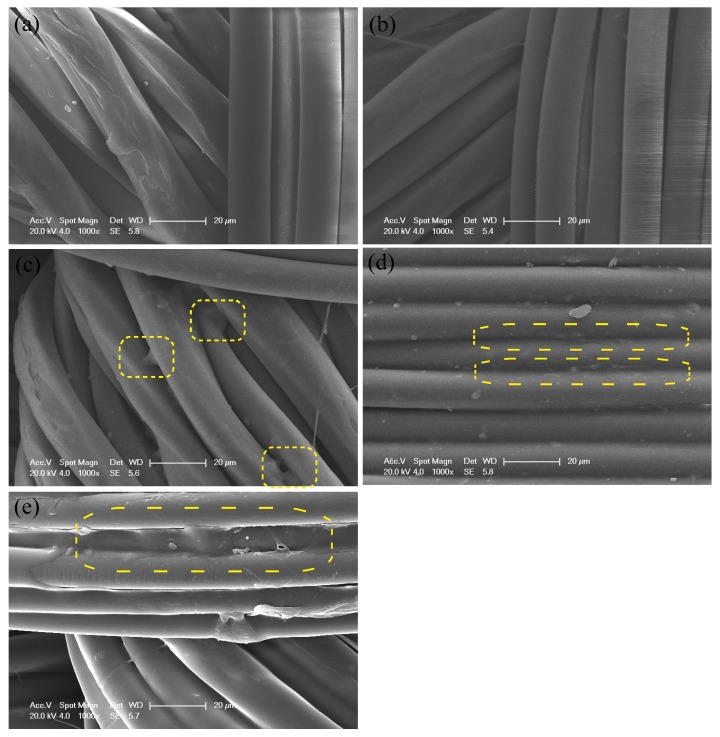
SEM images of Poly(ethyleneterephthalate) (PET) before (**a**) and after deposition of 1 bilayer (**b**); 3 bilayers (**c**); 5 bilayers (**d**) and 7 bilayers (**e**).

In order to mediate the dyeing property of PET fabric, the electrostatic layer-by-layer self-assembly technology was introduced. Polyelectrolyte PDADMAC and PAA were deposited onto the alkaline-treated PET fabric alternatively to form polyelectrolyte (PE) multilayer (PDADMAC/PAA). Figure 2 shows the SEM images of PET fabrics deposited with 1, 3, 5, and 7 bilayers of PDADMAC/PAA, respectively. It can be seen that the smooth morphology was well maintained for PET fabric before and after PE multilayers deposition. There was no obvious difference between alkaline-treated PET fabric (Figure 2a) and 1 bilayer PE multilayer-deposited PET fabric (Figure 2b). Whereas increasing the number of PE multilayers, some adhesion between single PET fibers was observed (Figure 1c, yellow square selects), and this phenomenon was more significant when 5 bilayers of PE multilayers deposited on PET fabric (Figure 2d). When the number of PE bilayers was increased to 7, gaps between each fiber were blocked and an obvious membrane-like structure was observed (Figure 2e, yellow square selects), similar to what we have obtained in previous work [25]. In addition, the hand feeling of the fabric worsened with the increase of PE multilayer deposition cycles, namely the fabric was getting hardened due to the deposition of PE multilayers. This might be because that during the process of LbL self-assembly, the PE multilayers are not only deposited onto the surface of each individual fiber but also deposited onto the fiber junction site. With the increase of PE deposition cycles, the PE multilayer films ultimately fill the limited space among the fibers [31]. Considering the vapor permeability and hand feeling of the fabric, 3 bilayers of PDADMAC/PAA were selected as maximum to deposit onto PET fabric for the dying process. 

**Figure 3:**
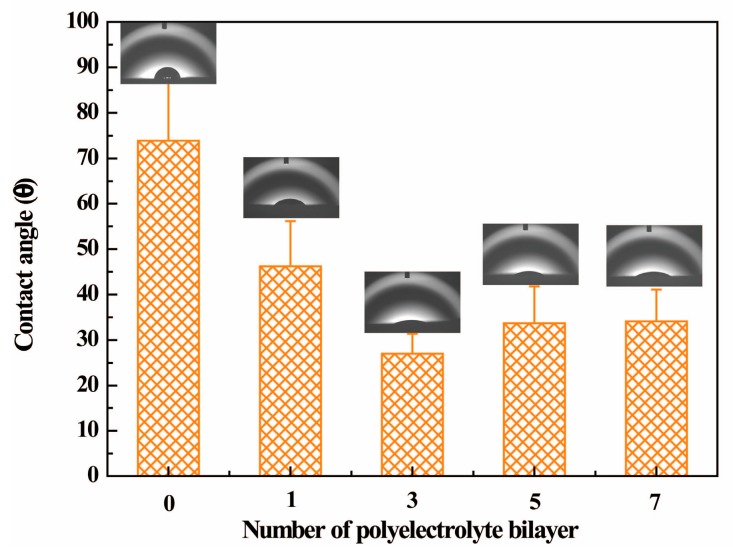
Contact angle of PET fabric with different bilayers of polyelectrolyte (PE) multilayer.

The effects of the number of bilayers on surface wettability of PET fabrics can be observed from the wetting measurements. Figure 3 shows the water contact angles for PET fabric with different treatment. Evidently the water absorptivity is gradually enhanced when the number of bilayers is less than 3. The contact angle decreased to 27.0° when PET fabric was deposited with 3 bilayers of PE multilayers, 23.5° lower than that of alkaline-treated PET fabric (50.5°). However, the contact angle of PET fabrics deposited with 5 bilayers of PE multilayer showed a slight increase, the contact angle was up to 33.7° and then reached a plateau when PET fabric deposited with 7 bilayers PE multilayer, indicating the wettability of the fabrics was saturated and the compact network structure of PE membrane might also hindered the further adsorption of more water (the PE membrane between the PET fibers was shown in Figure 2) [32]. Moreover, when the PE multilayers were within 3 bilayers, the gap between fibers may also be beneficial for the adsorption of water dropped onto the surface of PE multilayer-deposited PET fabrics when measuring the contact angle. The images of the water drop applied to the surface of alkaline-treated PET fabric and PET fabrics deposited with different PE bilayers are illustrated in Figure 3 (inset), consistent with the water contact angle results. 

**Figure 4:**
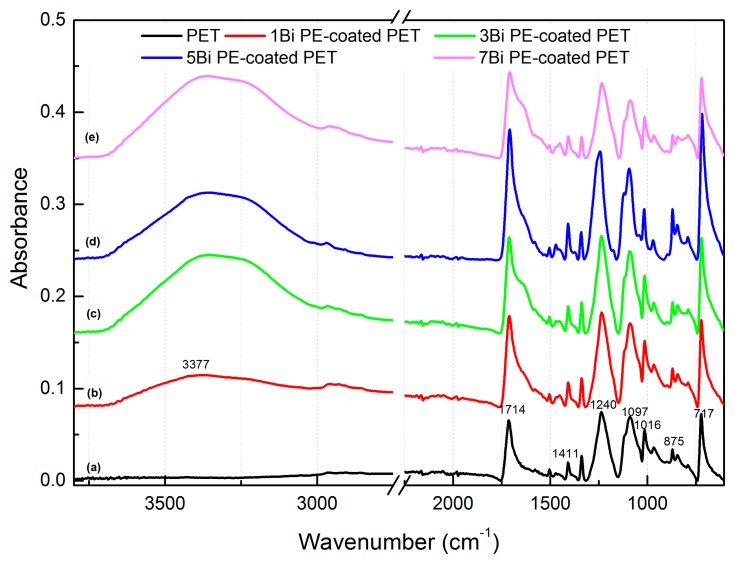
Fourier transform infrared (FTIR) spectra of PET fabric (a) and PET fabrics coated with (b) 1, (c) 3, (d) 5, and (e) 7 bilayers of PE multilayers, respectively.

FTIR ATR analysis was carried out to identify the spectroscopic changes between PE multilayer-deposited PET fabrics and original PET fabric surface, as shown in Figure 4. All the PE multilayer-deposited PET fabrics had a broad and protuberant adsorption peak at 3377 cm^−1^ assigned to the hydroxyl groups of PAA and H_2_O molecular adsorbed in the PE multilayers, which was not observed on the spectrum of the original PET fabric. This proved the successful deposition of PE multilayers on the PET fabrics. Except that, similar main peaks from 1714 cm^−1^ to 606 cm^−1^ were collected on the spectra of the original and PE multilayer-deposited PET fabrics. The strong peaks at 1714 cm^−1^ and 1240 cm^−1^ were corresponded to the stretch vibration of C=O and the stretch vibration of C–O bond of the PET fabric, respectively, which are consistent with the reported results in the literature [13]. It can be seen that the relative intensity of C=O band at 1714 cm^−1^ increased slightly for the PE multilayer-deposited PET fabrics, which possibly came from the –C=O stretching of dissociated carboxyl groups as a result of the PAA deposition onto PET fabrics. In addition, a peak at 1411 cm^−1^ was one of several assigned to an aromatic C–C stretching and –CH in-plane bending, a peak at 1097 cm^−1^ was assigned to C–O trans-vibration, and peaks at 1016 cm^−1^ and 875 cm^−1^ were assigned to aromatic =C–O bending and aromatic –CH in-plane bending, respectively [33].

**Figure 5:**
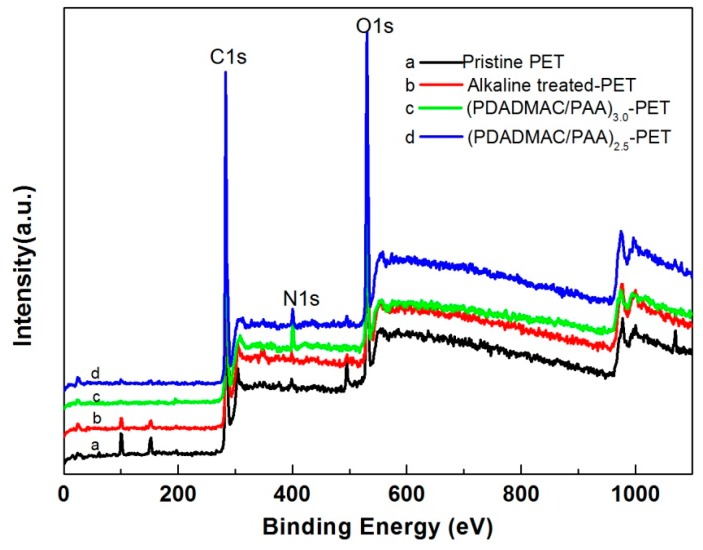
X-ray photoelectron spectroscopy (XPS) spectra of pristine fabric (a), alkaline treated fabric (b), (PDADMAC/PAA)_3.0_-PET fabric (c) and (PDADMAC/PAA)_2.5_-PET fabric (d).

**Figure 6:**
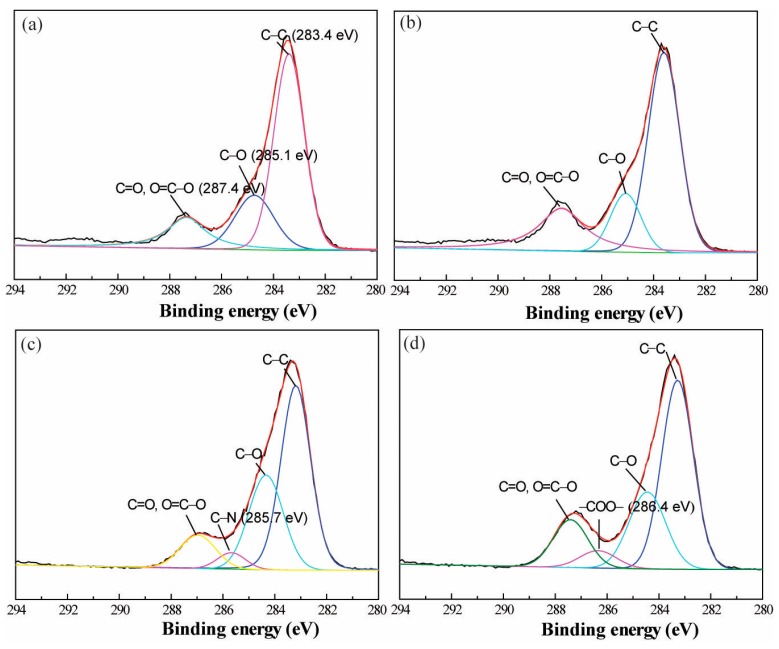
The simulated carbon (C1s) XPS analysis of pristine fabric (**a**); alkaline treated fabric (**b**); (PDADMAC/PAA)_3.0_-PET fabric (**c**) and (PDADMAC/PAA)_2.5_-PET fabric (**d**).

Furthermore, the XPS spectrum was analysed to identify the surface chemical composition (Figure 5). It indicates that the main elements of pristine PET and alkaline-treated PET fabrics are carbon and oxygen, the relative contents of C1s and O1s are shown in Table 1. XPS data showed a slight increase in the oxygen content (i.e., increases from 25.7% to 26.9%) and a regular decrease in carbon content (i.e., decreases from 73.5% to 72.5%) for the alkaline-treated PET fabric. This might be caused by the alkaline hydrolysis of PET fibers. It is interesting to note that a small amount of nitrogen was also detected on the pristine PET fabric and alkaline-treated PET fabric, respectively. This might be due to the contamination of nitrogen from the ambient air or, perhaps originate as a by-product from the complex formed due to partial interpenetration of the PDADMAC and PAA layers as a result of the LbL deposition process. For the (PDADMAC/PAA)_3.0_-PET fabric, the outermost layer of the PET fabric is predominantly PDADMAC, with some PAA and their complex (i.e., due to a degree of interpenetration of the oppositely charged layers that occurs in the LbL deposition method), thus the content of nitrogen significantly increased to 3.7%. In comparison, (PDADMAC/PAA)_2.5_-PET fabric showed a relatively low nitrogen content (2.3%) and a high oxygen content (30.7%) when the outmost layer of the deposition was predominantly PAA and some PDADMAC. Further, the presence of a small Cl2p signal at ~200 eV, likely results from the presence of PDADMAC. These results confirmed the successful deposition of PE multilayers onto the PET fabric. Peak fitting was applied to XPS spectra (Figure 6). Similar to the literature report [34], the carbon-containing groups on the PET fabric surface were ascribed to C–C (283.4 eV), C–O (285.1 eV), and C=O, and O=C–O (287.4 eV). In particular, the presence of C–N (285.7 eV) and –COO– (286.4 eV) on the spectra of (PDADMAC/PAA)_3.0_-PET fabric (Figure 6c) and (PDADMAC/PAA)_2.5_-PET fabric (Figure 6d) reinforced the successful deposition of polyelectrolytes on the PET fabric [35,36,37] respectively, which is absent in the original PET fabric. The peak areas of C1s spectra were also shown in Table 2. The intensity of C–N was 4.3% for the (PDADMAC/PAA)_3.0_-PET fabric and the intensity of –COO– increased to 5.7% for the (PDADMAC/PAA)_2.5_-PET fabric.

### 3.2. Coloration of PE Multilayer-Deposited PET Fabrics

**Figure 7:**
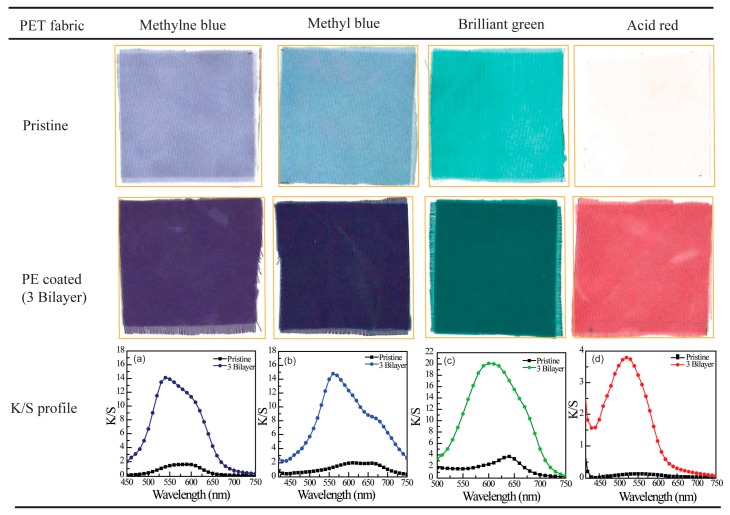
Photographs of pristine PET fabric and (PDADMAC/PAA)_3_-PET fabric dyed with different dyestuff. Profiles from (**a**) to (**d**) are the corresponding *K*/*S* profiles of each dyed PET fabrics.

The coloration of PE multilayer-deposited PET fabrics to water soluble dyestuffs was examined using four model dyes at room temperature, namely methylene blue, methyl blue, brilliant green and acid red. The photographs of dyed PET fabric samples and related *K*/*S* profiles were shown in Figure 7. Obviously, the 3 bilayers of PE multilayer-deposited PET fabrics show a darker color compared with that of pristine PET fabric for each tested dye, suggesting that modifying hydrophilic multilayer onto PET fabric could enhance the dye uptake of PET fabrics. This was also confirmed from their related *K*/*S* profiles shown in Figure 7a–d. In the case of methylene blue dyed (PDADMAC/PAA)_3_-PET fabric, a characteristic peak at 540 nm was collected, and the *K*/*S* value could reach up to 14.1; much higher than that obtained from the dyed pristine PET fabric (i.e., 1.26). Similarly, strong characteristic peaks were obtained on the methyl blue, brilliant green and acid red dyed (PDADMAC/PAA)_3_-PET fabrics, respectively, reinforcing the enhanced dyeability of PE multilayer-deposited PET fabrics. It is interesting to find that at the same dyeing condition, the light color was also observed on the corresponding methylene blue, methyl blue and brilliant green-colorized pristine PET fabrics. This might be due to the adhesion of dye molecules onto the surface and junction of PET fibers. However, the colorization of pristine PET fabric was almost impossible with acid dye, which might be related to the structure of dyes.

### 3.3. Effect of the Number of Bilayers on the Colorization of PE Multilayer-Deposited PET Fabrics

**Figure 8:**
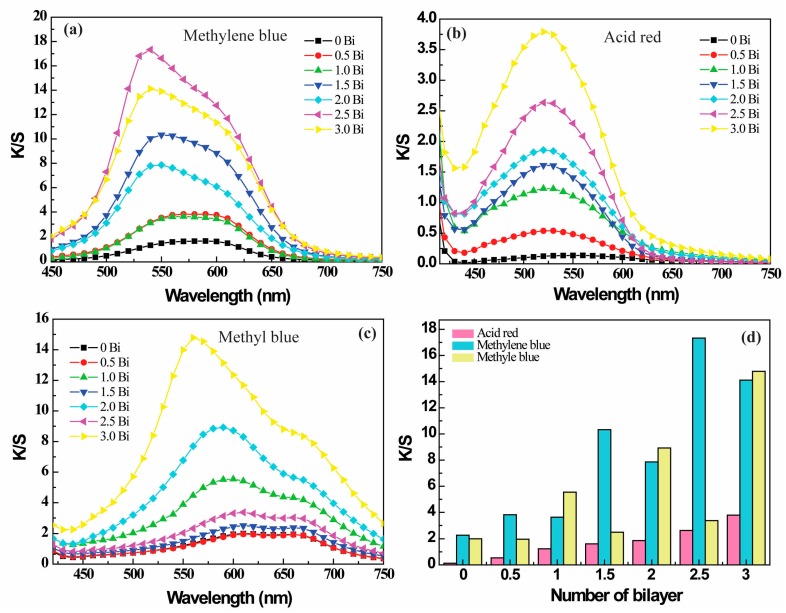
*K*/*S* profiles of different bilayer of PE-coated PET fabrics dyed with (**a**) methylene blue; (**b**) acid red; and (**c**) methyl blue; (**d**) *K*/*S* value of PE-coated PET fabrics as a function of the number of PE bilayer. (The surface of (PDADMAC/PAA)*_n_*-PET fabric was negatively charged when *n* = 0.5, 1.5, and 2.5. Alternatively, (PDADMAC/PAA)*_n_*-PET fabric was positively charged when *n* = 1, 2 and 3).

The effect of the number of bilayers on the colorization of PE multilayer-modified PET fabrics was further investigated. Figure 8 shows the *K*/*S* profiles of dyed PET fabrics deposited with different bilayers of PE multilayers. We saw that the *K*/*S* value at the characteristic peak increased with the number of bilayers of PE multilayer deposited onto the PET fabric. Moreover, the coloration capability of PE multilayer-deposited PET fabrics was dependent on the surface property of the fabric and the physical property of the dye (Figure 8d). Generally, the *K*/*S* value of PE multilayer-deposited PET fabrics was increased with the increasing of bilayer number. For methylene blue, the *K*/*S* values of PET fabrics deposited with 0.5, 1.5 and 2.5 bilayers of PE multilayers were higher than that obtained from corresponding PET fabrics deposited with 1.0, 2.0 and 3.0 bilayers of PE multilayers. However, in case of the methyl blue, the *K*/*S* value at the characteristic peak of PET fabrics deposited with 1.0, 2.0 and 3.0 bilayers of PE multilayers was significantly higher than that obtained from PET fabrics deposited with 0.5, 1.5 and 2.5 bilayers of PE multilayers, respectively. The *K*/*S* value of methyl blue dyed (PDADMAC/PAA)_1_-PET fabric (5.55) was even higher than that of (PDADMAC/PAA)_2.5_-PET fabric (3.37). For the acid red-dyed PE multilayer-deposited PET fabric, the *K*/*S* value showed a gradual increase with the deposition of PE multilayers. 

**Figure 9:**
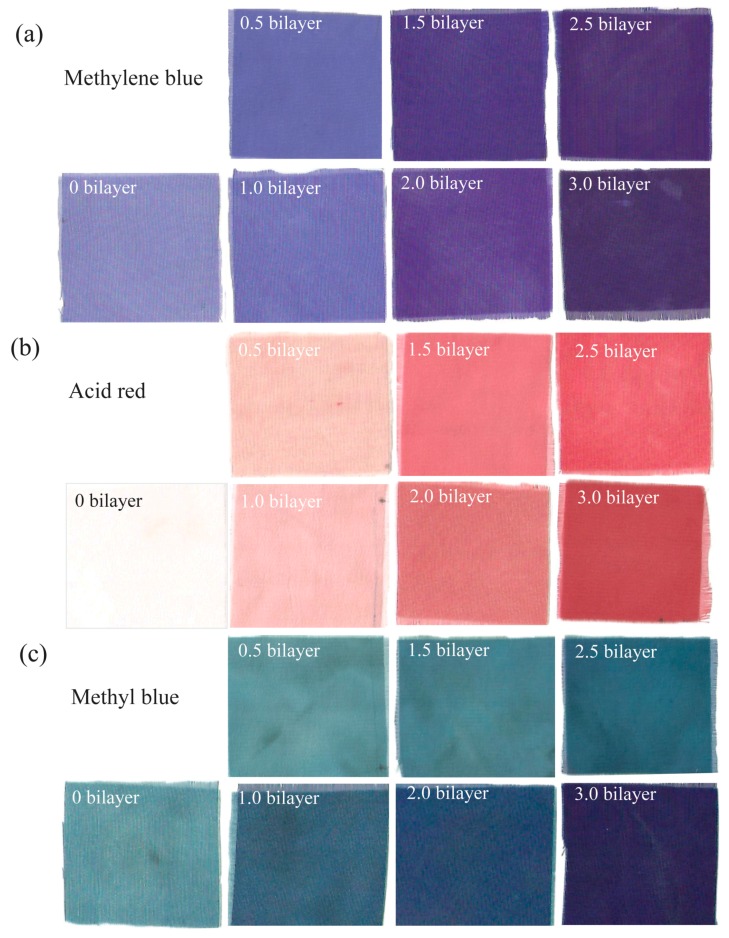
Photos of PET fabrics modified with different bilayer of PE membranes, (**a**) methylene blue; (**b**) acid red; and (**c**) methyl blue.

As for the coloration difference of PE multilayer-deposited PET fabrics, we think that the coloration capability of PE multilayer-deposited PET fabrics is mainly dependent on their surface property. For the half bilayer PE multilayer-deposited PET fabrics (the number of bilayer was 0.5, 1.5 and 2.5), the outermost layer of PE multilayers was PAA, which was negatively charged because of its ionization in aqueous solution. Methylene blue is water soluble, alkaline dyestuff. They can be positively charged in water and adsorbed on the negatively charged PET fabric surface through the electrostatic adsorption. Alternatively, when the outmost layer of the PE multilayer deposited on the PET fabric was PDADMAC, the positively charged PDADMAC would hinder the approaching of positively charged dye molecules because of electrostatic repulsion. In this scenario, the *K*/*S* values of PE multilayer-deposited PET fabrics dyed with methylene blue followed the order of (PDADMAC/PAA)_2.5_-PET > (PDADMAC/PAA)_3.0_-PET > (PDADMAC/PAA)_1.5_-PET > (PDADMAC/PAA)_2.0_-PET > (PDADMAC/PAA)_0.5_-PET > (PDADMAC/PAA)_1.0_-PET > PET fabric. Conversely, the positively charged PDADMAC could be benefit for the absorption of the negatively charged methyl blue. The *K*/*S* values of multilayer deposited PET fabrics followed the order of (PDADMAC/PAA)_3.0_-PET > (PDADMAC/PAA)_2.0_-PET > (PAA/PDADMAC)_1.0_-PET > (PDADMAC/PAA)_2.5_-PET > (PDADMAC/PAA)_1.5_-PET > (PDADMAC/PAA)_0.5_-PET ≥ PET fabric. Similarly, the positively charged PET fabric surface was also benefit for the adsorption of anionic acid red dye, following the order of (PDADMAC/PAA)_3.0_-PET > (PDADMAC/PAA)_2.5_-PET > (PDADMAC/PAA)_2.0_-PET > (PDADMAC/PAA)_1.5_-PET > (PDADMAC/PAA)_1.0_-PET > (PDADMAC/PAA)_0.5_-PET ≥ PET fabric. The different coloration trends of PE multilayer-deposited PET fabrics might be due to the synergistic action of surface hydrophilicity and polarity of modified PET fabric, the dye molecular and the dye polarity [36,38,39,40]. In addition, we note an obvious blue shift at the characteristic peak for the methylene blue dyed PET fabrics, with the increase of the bilayer number of PE multilayers deposited onto the fabrics (Figure 8a), which was not observed on the *K*/*S* profiles of acid red dyed samples (Figure 8b). In case of methyl blue dyed samples, the slight blue shift at the characteristic peak on the *K*/*S* profiles only happened on the integral bilayer PE multilayer-deposited PET fabrics (Figure 8c). In regard to this, the mechanism was not clear, which might be related to the structures of dye molecular and properties of polyelectrolytes deposited onto the surface of PET fabric. Figure 9 presents the photographs of PE multilayer-deposited PET fabrics after coloration with methylene blue, acid red and methyl blue, respectively. It is clear that the bright blue color was increased for the PE multilayer-deposited PET fabrics after dyeing with methylene blue (basic dye) and PET fabric deposited with half bilayer of PE multilayers shows a slight darker blue color compared with that obtained at integral bilayer of PE multilayer-deposited PET fabrics (Figure 9a), reinforcing the results that were shown in Figure 8a. For the acid red dyed PE multilayer-deposited PET fabrics, a gradual color increase was observed (Figure 9b), consistent with results shown in Figure 8b. Similarly, the stone color of PE multilayer-deposited PET fabrics after dyeing with methyl blue displayed a gradual increase with the increase of PE multilayers, whereas PET fabric deposited with integral bilayer of PE multilayers shows an obviously darker color compared with that obtained at half bilayer of PE multilayer-deposited PET fabrics (Figure 9c). 

**Figure 10:**
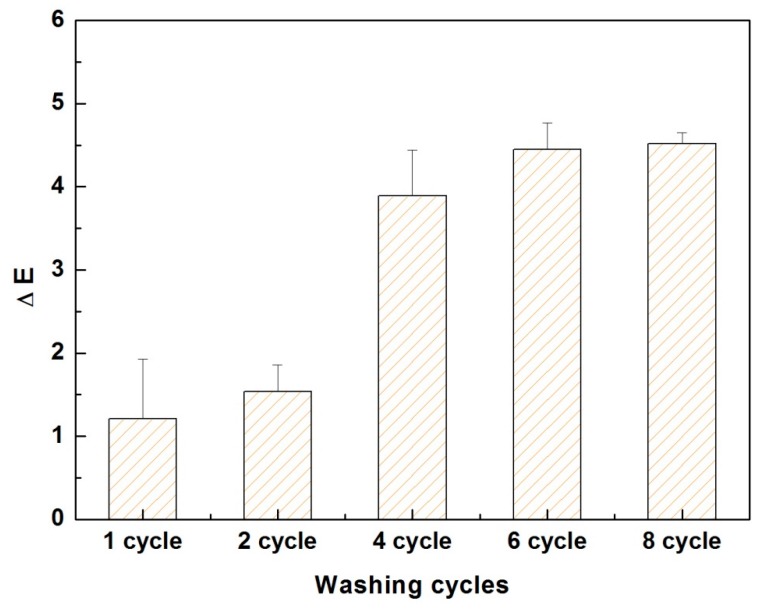
Evolution of the color difference (∆*E*) of methylene blue dyed (PDADMAC/PAA)_2.5_-PET fabric with increasing the number of washing cycles.

It is known that textiles are subjected to frequent washing and exposure to sunlight during their use. Hence, the permanency of the bond between the dye molecules and the surface of PE multilayer-deposited PET fabric was investigated by colorfastness tests. The (PDADMAC/PAA)_2.5_-PET fabrics dyed with the methylene blue (i.e., 10 g/L) was washed for 45 min in the presence of ECE reference detergent at 40 °C under each washing cycles. The color differences (∆*E*) of fabrics before and after washing were shown in Figure 10. ∆*E* is a perceptual colour difference calculation method especially designed for textiles and accurately quantifies the extent to which the two coloured textile specimens match. A value of ≤1.0 signifies an imperceptible difference in color. Whilst the color difference with ∆*E* more than 2.0 was reported to be perceived correctly 100% of the time by human observers and increasing ∆*E* values indicate greater deviation and difference from the reference sample [41]. The average ∆*E* value of fabric was measured to be 1.21 after the first washing cycle, and then slightly increased to 1.54 after second washing, suggesting that there was no noticeable color difference between dyed PET fabrics before and after two washing cycles [41]. The ∆*E* value increased to 4.5 after 6 washing cycles and remained stable with further increase in number of washing cycles. According to the ∆*E* vale, the gray scale rating for color change after eight washing cycles was assessed to be 3.0, demonstrating that the PE multilayer-deposited fabrics after dyeing with methyl blue have good washing fastness. 

**Figure 11:**
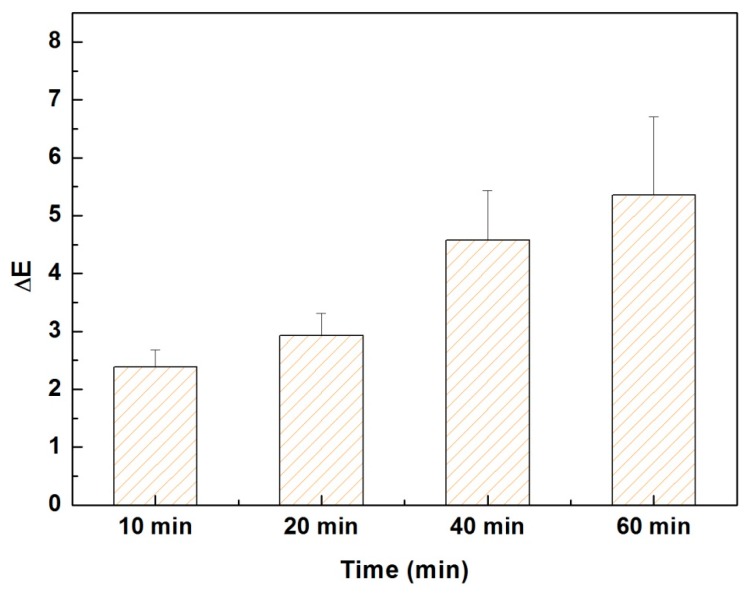
Evolution of color difference (∆*E*) of methylene blue dyed (PDADMAC/PAA)_2.5_-PET fabric with increasing time after exposure in UV light.

It has been well established that the color-fading of textile under the sunlight is mainly attributed to the UV light-degradation of the chemical structures of the dyes. Therefore, the evaluation of color-fading of fabrics under the sunlight is quite important in textile industry. Herein, the color-fading of methyl blue dyed (PDADMAC/PAA)_2.5_-PET fabric after exposure to UV light (BZGY 908 standard UV light box) for different periods were studied, shown in Figure 11. Clearly the color difference (∆*E*) of dyed PET fabrics was gradually increased with the prolongation of exposure time in UV light. The ∆*E* value reached up to 5.35 when the exposure time was up to 60 min. These results indicated that (PDADMAC/PAA)_2.5_-PET fabric with coloration of methylene blue have a moderate color fastness even after a long time exposure of UV light, but more work also needs to do to improve the anti-UV property for the modified PET fabrics developed in this study.

## 4. Conclusions

In summary, we have successfully developed a facile approach to producing hydrophilic PET fabric with tuneable surface properties. The PE multilayers deposited onto PET fabrics through the LbL electrostatic self-assembly method allow for its improved coloration with the model cationic (methylene blue and brilliant green) and anionic dyestuffs (methyl blue and acid red) within 2 h at room temperature and the normal pressure. Systematic investigation demonstrated that the coloration of PE multilayer-deposited PET fabrics was strongly dependent on the surface property of fabrics and the physical property of dyestuffs. The remarkable coloration capability of PE multilayer-deposited PET fabrics is mainly attributed to the hydrophilic groups such as the amino group of PDADMAC and carboxylic acid group of PAA deposited on PET fabric, which increase the substantivity to acid dyes and basic dyes via complementary electrostatic attraction. Modification of PET fabric surface using LbL self-assembly may find numerous applications to overcome other problems related to PET coloration and functionalization of PET fabrics. 

## Figures and Tables

**Table 1:** Relative surface chemical composition of different PET fabrics.

Sample	Chemical Composition of Surface (%)			
	C1s	O1s	N1s	O1s/C1s
Pristine PET	73.5	25.7	0.8	34.9
Alkaline-t-PET	72.5	26.9	0.6	37.1
(PDADMAC/PAA)_3.0_-PET	74.7	21.6	3.7	28.9
(PDADMAC/PAA)_2.5_-PET	67.0	30.7	2.3	45.8

**Table 2:** The relative peak areas of C1s spectrum of different PET fabrics.

Sample	Relative Area Corresponding to Different Chemical Bonds (%)				
	C–C	C–O	C=O, O–C=O	C–N	–COO–
Pristine PET	60.4	21.9	17.7	/	/
Alkaline-t-PET	58.1	16.0	25.9	/	/
(PDADMAC/PAA)_3.0_-PET	54.0	30.5	11.1	4.3	/
(PDADMAC/PAA)_2.5_-PET	53.8	25.3	15.1	/	5.7

Note: /, not applicable.
